# Does Language Influence the Vertical Representation of Auditory Pitch and Loudness?

**DOI:** 10.1177/2041669517716183

**Published:** 2017-06-23

**Authors:** Irune Fernandez-Prieto, Charles Spence, Ferran Pons, Jordi Navarra

**Affiliations:** Fundació Sant Joan de Déu, Parc Sanitari de Sant Joan de Déu, Esplugues de Llobregat (Barcelona), Spain; Crossmodal Research Laboratory, Department of Experimental Psychology, University of Oxford, Oxford, UK; Institute of Neurosciences, University of Barcelona, Barcelona, Spain; Crossmodal Research Laboratory, Department of Experimental Psychology, University of Oxford, Oxford, UK; Department of Cognition, Development and Educational Psychology, University of Barcelona, Barcelona, Spain; Institute of Neurosciences, University of Barcelona, Barcelona, Spain; Institut de Recerca Pediàtrica, Hospital Sant Joan de Déu, Barcelona, Spain; Department of Cognition, Development and Educational Psychology, University of Barcelona, Barcelona, Spain; Fundació Sant Joan de Déu, Parc Sanitari de Sant Joan de Déu, Esplugues de Llobregat (Barcelona), Spain

**Keywords:** crossmodal correspondences, language, spatial elevation, pitch, loudness

## Abstract

Higher frequency and louder sounds are associated with higher positions whereas lower frequency and quieter sounds are associated with lower locations. In English, “high” and “low” are used to label pitch, loudness, and spatial verticality. By contrast, different words are preferentially used, in Catalan and Spanish, for pitch (high: “agut/agudo”; low: “greu/grave”) and for loudness/verticality (high: “alt/alto”; low: “baix/bajo”). Thus, English and Catalan/Spanish differ in the spatial connotations for pitch. To analyze the influence of language on these crossmodal associations, a task was conducted in which English and Spanish/Catalan speakers had to judge whether a tone was higher or lower (in pitch or loudness) than a reference tone. The response buttons were located at crossmodally congruent or incongruent positions with respect to the probe tone. Crossmodal correspondences were evidenced in both language groups. However, English speakers showed greater effects for pitch, suggesting an influence of linguistic background.

Many studies have suggested the existence of crossmodal correspondences between specific acoustic features such as pitch or loudness (i.e., high vs. low sounds) and other perceptual features such as spatial elevation (high vs. low positions, respectively; see also Deroy, Fernández-Prieto, Navarra, & Spence, in press; Spence, 2011, for reviews). These crossmodal interactions between pitch and spatial elevation often generate *congruency effects*. Faster and more accurate responses to high or low sounds are observed when these stimuli are combined with other stimuli presented in upper or lower spatial positions, respectively.

In one study, Rusconi, Kwan, Giordano, Umilta, and Butterworth (2006) reported crossmodal effects between pitch and spatial elevation. Participants made speeded pitch discrimination responses comparing the frequency of a probe and a reference tone by pressing one of two different keys (for “higher” or “lower” responses) on a computer keyboard. The results revealed that participants’ responses to “higher” and “lower” tones were faster and more accurate when they had to press a button located at a “symbolically” upper position (the “6” key) or at a lower position (spacebar), respectively. Thus, the reaction time (RT) was modulated by the spatial location of the response button in a simulated vertical axis. Similar results were found by Puigcerver, Gómez-Tapia, Rodríguez-Cuadrado, and Navarra (2016), who investigated the crossmodal correspondence between loudness and spatial elevation. In this study, participants judged the intensity of a probe tone with respect to a reference tone. This time, the participants responded using two keys that were physically located above and below a rest platform. The results indicated that spatial elevation is not only associated with pitch but also with loudness (see also Marks, 1987).

According to linguistic relativity (also known as the Sapir–Whorf hypothesis; Sapir, 1929; Whorf, 1956), the semantic diversity of our native languages induce differences in our perception and cognition. Previous studies have demonstrated that linguistic experience modulates several aspects of cognitive and perceptual systems (see Lupyan, 2012, for a review). For instance, language has been shown to influence recognition memory (Lupyan, 2008), simple visual detection (Lupyan & Spivey, 2010), motion perception (Meteyard, Bahrami, & Vigliocco, 2007), and the temporal perception of audiovisual signals (Navarra, Alsius, Velasco, Soto-Faraco, & Spence, 2010). An interesting issue that still remains unresolved refers to the possibility that the link between spatial elevation and auditory features such as pitch or loudness may be influenced by the activation of a common linguistic or metaphoric code (see Casasanto, 2014, for a review). Indeed, most cultures symbolically represent acoustic pitch vertically (e.g., musical notation) since ancient times, for example, in the Seikilos epitaph (AD 100), where the ascending frequencies appear engraved in higher spatial positions. This metaphorical representation is also observed in a common vocabulary for both dimensions. For example, the words “high” and “low” in English activate both auditory and spatial concepts. The use of space-centered metaphorical expressions to refer to auditory features was already suggested by the philosopher Carl Stumpf late in the 19th century. Stumpf (1883) pointed out that sounds are usually defined with linguistic labels referring to high- and low-spatial positions in the majority of languages.

Romance languages such as Spanish, Catalan, and French generally use linguistic labels that do not provide spatial information to describe pitch. However, languages such as Turkish, Farsi (or Persian), and Zapotec use terms related to thickness in order to refer to acoustic frequencies: While “thin” is associated with high frequencies, “thick” is associated with low frequencies (see Dolscheid, Shayan, Majid, & Casasanto, 2013; Shayan, Oztur, & Sicoli, 2011). Crossmodal correspondences between pitch and spatial elevation can be observed in speakers of languages that use terms to label pitch that do not refer to any spatial feature. In a study by Parkinson, Kohler, Sievers, and Wheatley (2012), participants from a linguistically isolated Cambodian hill tribe, whose language does not contain spatial linguistic labels to describe pitch, showed the perceptual association between pitch and spatial elevation just as participants whose language used spatial terms to describe the frequency of sounds.

Other evidence suggesting that crossmodal correspondences can occur without any language modulation comes from studies conducted with prelinguistic infants (Dolscheid, Hunnius, Casasanto, & Majid, 2014; Fernández-Prieto, Navarra, & Pons, 2015; Walker et al., 2010). For example, in Walker et al.’s (2010) study, 3- to 4-month-old infants tended to look longer at a visual stimulus that moved upwards or downwards coherently with respect to a simultaneously presented sound that progressively changed in pitch (but see Lewkowicz & Minar, 2014). The evidence presented so far indicates that the crossmodal correspondence between spatial elevation and pitch emerges without any influence from language labelling (see also Lewkowicz & Turkewitz, 1980). It is possible that the infants’ exposure to statistical regularities in the environment strengthen audiovisual crossmodal correspondences. For example, higher and lower frequency sounds are generally transmitted from sources that are higher and lower in space, respectively (Parise, Knorre, & Ernst, 2014). However, an unsolved question refers to the possibility that the use of the same descriptor to label two different perceptual attributes associated with different sensory modalities can modulate crossmodal associations.

The aforementioned crossmodal correspondences could take place not only at a perceptual but also at a higher level such as semantic processing level (Ben-Artzi & Marks, 1999; Melara & Marks, 1990; Sadaghiani, Maier, & Noppeney, 2009). Melara and Marks (1990) reported congruency effects using a Garner-type interference paradigm involving linguistic stimuli. Participants discriminated the words “high” and “low” more rapidly when they were presented together with a high- or a low-pitched sound, respectively. This Garner interference occurred between the pitch and the word’s meaning. The authors concluded that the same semantic concepts were activated during the processing of both the words and the sounds having a different pitch.

If the crossmodal association between specific auditory features (e.g., pitch or loudness) and spatial elevation is mediated by language, different outcomes should be expected when one’s language shares the same linguistic terms used to describe these auditory features and verticality. To test this hypothesis, the performance of a group of English and Spanish/Catalan speakers, whose languages differ in terms of the spatial connotation of the words used to denote pitch and spatial elevation, was compared. The participants performed a speeded pitch and loudness discrimination task in which they had to compare the frequency or loudness of a probe and a reference tone by pressing one of two different buttons located above (“up” position) or below (“down” position) a rest/starting position. In English, the words “high” and “low” are used to define pitch, loudness, and verticality. By contrast, different words are used, in Catalan and Spanish, to represent pitch (“agut” -high- vs. “greu” -low-, in Catalan, and “agudo” -high- vs. “grave” -low-, in Spanish) and verticality (“alt” -high- vs. “baix” -low-, in Catalan, and “alto” -high- vs. “bajo” -low-, in Spanish). Interestingly, the words that represent verticality are also used to represent loudness in both Catalan and Spanish. Therefore, if crossmodal correspondences are influenced by language, we should observe (a) similar congruency effects for loudness and verticality in both English and the Catalan/Spanish speakers but (b) less congruency effects between pitch and verticality in the latter group.

## Results

We compared the performance of participants discriminating pitch and loudness between a probe and a reference tone in congruent and incongruent conditions in the English and the Spanish/Catalan group. The average RTs in correct trials and the total number of errors were selected as dependent measures. RTs faster than 200 ms (anticipatory responses) were not included in the statistical analyses (<0.5% of the total of trials).

### Pitch analyses

#### Reaction times

**Figure 1.**
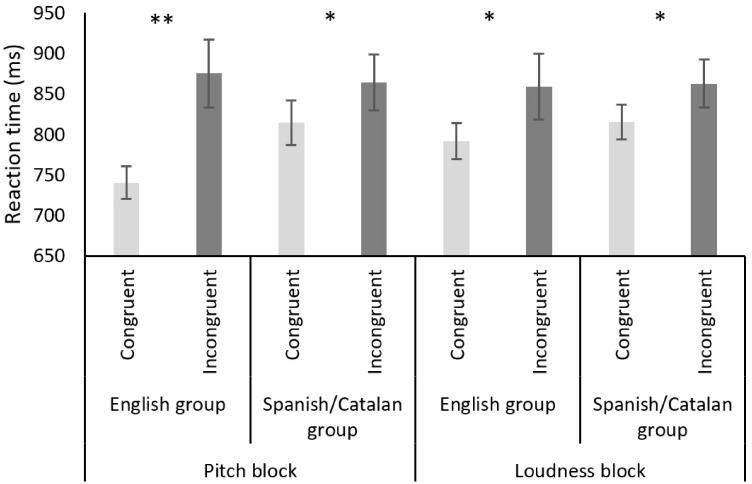
Mean RTs (in milliseconds) in each block (pitch/loudness) and group (English/Spanish–Catalan) for the two conditions (congruent/incongruent). Error bars indicate the standard error of the mean. Single and double asterisks indicate a significant difference between conditions (*p* < .05, and *p* < .01, respectively).

A mixed, repeated measures analysis of variance (ANOVA) including “Congruence” (congruent vs. incongruent) as the within-participants factor and “Musical Expertise” (Musicians vs. Non-musicians) and “Linguistic Group” (English vs. Spanish/Catalan) as between-participants factors revealed only a significant interaction between “Linguistic Group” and “Congruence” factors, *F*(1, 47) = 5.514, *p* = .023, η_p_^2 ^= .105. A significant main effect of congruence was found, *F*(1, 47) = 18.557, *p* < .001, η_p_^2 ^= .283. Pairwise *t*-tests (two-tailed) revealed significantly faster RTs in the congruent than in the incongruent condition in both the English (*t*(26) = 4.072, *p* < .001, Cohen’s *d* = .794) and the Spanish/Catalan group (*t*(23) = 2.076; *p* = .049, Cohen’s *d* = .326) (see Figure 1).

**Figure 2.**
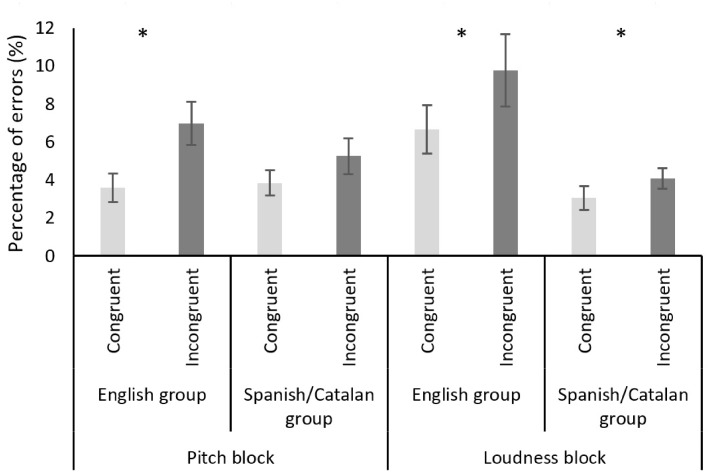
Mean percentage of errors in each block (pitch/loudness) and group (English/Spanish–Catalan) for the two different conditions (congruent/incongruent). Error bars indicate standard error of the mean. Single asterisk indicates a significant difference between conditions (*p* < .05).

#### Errors

A subsequent ANOVA was conducted with the total number of errors including “Congruence” as the within-participants factor and “Linguistic Group” and “Musical Expertise” as between-participants factors. The analysis revealed only a significant main effect of congruence (*F*(1, 47) = 10.584, *p* = .007, η_p_^2 ^= .184) but no interactions (all *p* > .1) (see Figure 2).

### Loudness analyses

#### Reaction times

An ANOVA including “Congruence” as a within-participants factor and “Linguistic Group” and “Musical Expertise” as between-participants factors revealed no interaction between them (all *p* > .1). Again, a significant effect of Congruence was found (*F*(1, 47) = 7.932, *p* = .002, η_p_^2 ^= .144) (see Figure 1).

#### Errors

An ANOVA including the within-participants factor “Congruence Errors” and the between-participants factors “Linguistic Group” and “Musical Expertise” did not reveal any interaction between them (all *p*s > .1). General congruency effects were observed, *F*(1, 47) = 4.167, *p* = .047, η*_p_*^2 ^= .081 (see Figure 2).

## Discussion

The results of the present study suggest that auditory pitch and loudness can modulate spatial processing (see Spence, 2011, for a review). The effects observed here were similar to those previously found by Rusconi et al. (2006) and Puigcerver et al. (2016). The two linguistic groups tested here exhibited crossmodal correspondences between spatial elevation and either pitch or loudness. Their responses were faster and more accurate in the congruent trials (i.e., responding to a higher pitch with the upper key) than in the incongruent trials in both auditory tasks (pitch and loudness). However, a greater difference between the congruent and the incongruent condition was observed in the English group when judging pitch than in the Catalan/Spanish group. This result could be interpreted as a consequence of the English speakers having a stronger association between pitch and spatial elevation than the Spanish/Catalan speakers. It is important to note that the same words are used in English to refer to both acoustic pitch and verticality. As a result, another representational link between the tested perceptual features could be present in English but not in Spanish/Catalan speakers.

Indeed, although the Spanish and Catalan words for “high” (“alto/alt”) and “low” (“bajo/baix”) are rarely used to define acoustic pitch, the words “agudo/agut” and “grave/greu”, with no vertical connotation, are extensively used instead. Note, though, that “alto/alt” and “bajo/baix” are both used for verticality and loudness in these two languages. According to the hypothesis suggesting that the crossmodal correspondence between verticality and specific acoustic features can be modulated by the perceiver’s linguistic background, similar results were expected for the association between loudness and verticality in the two linguistic groups. To be clear, the results confirmed this hypothesis: No differences in loudness discrimination were found between the English and the Spanish/Catalan speakers. Note that English, Catalan, and Spanish use terms associated with spatial elevation to define the loudness. Consequently, the same linguistic metaphorical labels are used for auditory and spatial features.

Speakers of English use the same linguistic terminology to refer to loudness, pitch, and spatial elevation. Therefore, these auditory terms could activate mental representations of space during the performance of pitch- and loudness-based judgments. As stated by Lakoff and Johnson (1980), equivalent terms used to label characteristics of two different dimensions could influence the way to conceptualize these characteristics. That is, when an English speaker uses the term “high” to define the frequency or the intensity of a sound, a mental representation of elevation in the space might be activated at the same time.

Although the current results show that the English speakers exhibit a stronger association between pitch and verticality than Spanish/Catalan speakers, this result cannot be taken as evidence that language is indispensable for this association to occur. In fact, Spanish/Catalan speakers also showed a pitch–spatial elevation association, albeit less robustly. As shown previously, the emergence of crossmodal correspondences can occur before the acquisition of language (see Walker et al., 2010) or even in nonhuman animals (for example, chimpanzees; see Ludwig, Adachi, & Matzuzawa, 2011). Several authors suggest that some of these perceptual associations may be based on the adaptation to the statistics of the natural environmental. For instance, Parise et al. (2014) demonstrated, by directly recording and measuring several acoustic features, that the association between frequency and spatial elevation could be based on universal statistics from natural auditory scenes in which higher frequencies are originating from higher positions in space. Since these correlations are derived from the experience in the environment, no mediation via language would be necessary for this crossmodal correspondence to surface (see Lakoff & Johnson, 1980).

However, even though language does not seem essential for the crossmodal association between pitch and verticality, the fact that English uses spatial linguistic terms to define acoustic pitch may strengthen these perceptual mappings. Interestingly, the association between pitch and spatial elevation seems to arise at the basic level of perceptual information processing where language is not required but also at higher levels of processing where language is indispensable, for example at a semantic level (see Ben-Artzi & Marks, 1999).

At a speculative level, one possibility might be that English speakers process these crossmodal associations at two different levels: perceptual and semantic; while the Spanish/Catalan speakers process pitch-spatial elevation association only at the perceptual level.

In conclusion, the results of the present study show that auditory features (e.g., loudness and pitch) can modulate visuospatial processing. According to previous literature, crossmodal correspondences between pitch, loudness, and spatial elevation occur automatically (see Parise et al., 2014). However, language seems to strengthen these associations. Due to the use of the same metaphorical linguistic labels for different sensory features (e.g., “high” to define a visuospatial and an auditory feature), language could perhaps facilitate these natural crossmodal correspondences.

## Methods

### Participants

In the current study, the inclusion criterion for musicians was to have musical experience as a professional, music student, or high-level amateur for a minimum of 4 years. According to the language questionnaire, none of the participants was bilingual in English and any Romance language (e.g., French or Spanish).

Twenty-seven native monolingual speakers of English (21 female, mean age 23.1 ± 4.2 years, two left-handed) and 24 native speakers of Catalan and Spanish (19 female, mean age 19.7 ± 2.8 years, two left-handed; 22 native bilingual Catalan/Spanish speakers and 2 Spanish monolingual speakers) participated in the experiment and were tested at the University of Oxford and the University of Barcelona, respectively.

The participants reported normal or corrected-to-normal vision and normal hearing. They received 5 pounds or 5 euros for participating in the study. Written informed consent was obtained from all of the participants before taking part in the experiment. The study was approved by the Central University Research Ethics Committee at the University of Oxford (MS-IDREC-C1-2015-212) and and the Hospital Sant Joan de Deú Ethics Committee.

### Apparatus

An Intel Core® laptop computer with a 15-in. monitor (HP Pavilion, China; refresh rate = 60 Hz) and headphones (Phillips SHP1900, China) were used for the experiment for all of the participants. The experiment was run using E-Prime 2.0 (Psychology Software Tools Inc., Pittsburg, PA) in a quiet and dimly lit room. The participants sat approximately 60 cm from the monitor screen.

**Figure 3.**
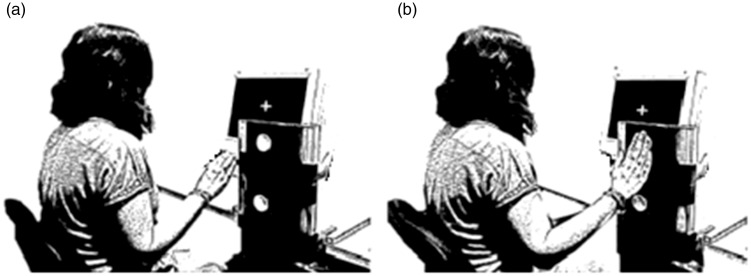
Experimental setup. (a) Participants had to place their hand on the starting position, a platform located between the two response buttons. (b) Participants responded whether a probe pitch was higher or lower (in pitch or loudness) than the reference tone.

The participants’ responses were obtained by means of a modified computer keyboard that consisted of a flat panel with two response foam buttons located above and below a rest platform (see Figure 3). The distance between the two response buttons was 15.7 cm and the rest platform was located at the same distance with respect to each of the two buttons, on the left side of the response board.

### Procedure

Before the experiment, all participants completed a language questionnaire to assess their usage of a specific language(s). The language questionnaire assessed the participant and his/her family places of birth, as well as the languages used in their everyday life and the age of acquisition. In addition, the level of auditory and reading comprehension, oral communication (fluency and pronunciation), and writing of the language or languages was evaluated.

The methods used previously by Puigcerver et al. (2016) were adapted in the current study. The experiment had two independent blocks (pitch and loudness) with two independent conditions (congruent and incongruent). Blocks and conditions were randomized across participants.

#### Pitch block

Each trial began with the appearance of a white fixation cross at the center of the screen for 250 ms. Next, the 261 Hz reference tone was presented for 1120 ms. This was followed by a random appearance of one of the eight different comparison probe tones (165, 185, 208, 233, 294, 330, 367, and 415 Hz). The participants had to judge as rapidly and accurately as possible whether the second tone was higher or lower than the first. Feedback (“correct,” “incorrect” or “no response detected”) was provided 750 ms after the participant’s response or after 3500 ms if no response was given.

The Pitch block consisted of two separated conditions (congruent and incongruent), each composed of 96 trials (12 trials for comparison tone). The block had a total of 192 trials. In the congruent trials, the participants responded to high and low tones with the upper and lower buttons, respectively. In the incongruent condition, the participants had to respond with a reversed pattern, that is, to high and low tones with the lower and the upper buttons, respectively.

The participants completed a training session before starting the main test blocks. These sessions consisted of a simplified version of the pitch block in which only two of the comparison tones (i.e., the ones with the lower and the higher tone; 165 Hz and 415 Hz) were used in 10 different trials (including 5 congruent and 5 incongruent trials presented randomly).

#### Loudness block

The procedure used in this block was identical to the pitch block, but all of the tones had the same pitch (261 Hz) but different loudness. In this block, the participants judged whether a probe tone (52, 55, 58, 61, 67, 70, 73, and 76 dB) was louder or softer than the reference tone (64 dB).

For the congruent condition, the participants responded to louder and quieter tones with the upper and lower button, respectively. In the incongruent condition, the participants responded to louder and softer tones with the lower and the upper buttons. The training session included two comparison tones: the highest (76 dB) and the lowest (52 dB) ones.
